# Serum progesterone, glycosylated hemoglobin and insulin levels with the risk of premature rupture of membranes in gestational diabetes mellitus

**DOI:** 10.1016/j.clinsp.2024.100461

**Published:** 2024-08-30

**Authors:** LiRong Zhou, XueSong Xiong, LianHua Chen

**Affiliations:** a Department of Endocrinology and Metabolism, Affiliated Sinopharm Dongfeng General Hospital, Hubei University of Medicine, Shiyan City, Hubei Province, China; b Department of Endocrinology, Ezhou Central Hospital, Ezhou City, Hubei Province, China; c Department of Nursing, Shiyan Renmin Hospital, The Affiliated People's Hospital of Hubei University of Medicine, Shiyan City, Hubei Province, China

**Keywords:** Gestational Diabetes Mellitus (GDM), Premature Rupture of Fetal Membranes (PROM), Progesterone, HbA1c, Insulin

## Abstract

**Objective:**

To discuss the correlation between serum progesterone, glycosylated Hemoglobin (HbA1c), and insulin levels in pregnant women with Gestational Diabetes Mellitus (GDM) and the risk of Premature Rupture of Membranes (PROM).

**Methods:**

A retrospective analysis was conducted on 52 patients diagnosed with GDM who also presented with PROM (Observation group) and compared with 89 patients diagnosed with GDM but not complicated with PROM (Control group). Progesterone, insulin, and HbA1c were detected. Risk factors for PROM in GDM patients were analyzed.

**Results:**

The observation group had higher HbA1c and fasting blood glucose levels. Poor blood glucose control and GWG are risk factors for PROM in GDM patients. PROM increases adverse pregnancy outcomes in GDM. HbA1c, insulin, and HOMA-IR can predict the risk of PROM in GDM.

**Conclusions:**

The effective prediction of preterm PROM can be achieved through the monitoring of serum HbA1c, insulin levels, and insulin resistance in patients with GDM.

## Highlights

• The observation group had higher HbA1c and fasting blood glucose levels. Poor blood glucose control and GWG are risk factors for PROM in GDM patients.
• PROM increases adverse pregnancy outcomes in GDM.
• HbA1c, insulin, and HOMA-IR can predict the risk of PROM in GDM.

## Introduction

Gestational Diabetes Mellitus (GDM) is a disease which women temporarily appear an abnormal elevation of blood glucose during pregnancy,^1^ and it is extremely harmful to the health of GDM patients and fetuses.^2^ However, the pathogenesis of GDM is still unknown. According to the current research and analysis, possible factors affecting the pathogenesis of GDM patients include insulin resistance, inflammatory factors, oxidative stress, genetic factors, adipocyte factors and so on.^3^ Premature Rupture Of Membrane (PROM) refers to the spontaneous rupture of the fetal membrane before birth.^4^ Premature Rupture Of Membranes (PROM) is a common pregnancy complication in the field of obstetrics, associated with risks such as intrauterine infection, fetal preterm birth, fetal hypoplasia, and increased perinatal mortality.^5^^,^^6^ The occurrence rate of maternal PROM during clinical pregnancy ranges from 2 % to 7 %.^7^ Research indicates a rising trend in the occurrence rate of premature rupture of fetal membranes among patients with GDM.^8^ Progesterone levels typically increase throughout pregnancy, and studies have shown a significant correlation between changes in progesterone levels and the initiation of delivery through membrane rupture.^9^ HbA1c can be used as an indicator to reflect the blood sugar level of GDM patients and is commonly applied to clinical treatment and diagnosis. Studies showed that the Hba1c levels are closely related to pregnancy outcomes.^10^ Fasting Insulin Level (FINS) is closely related to the body's ability to regulate blood sugar.^11^ The alterations in hormone levels observed in patients with Gestational Diabetes Mellitus (GDM) can induce insulin resistance, particularly evident during the mid to late stages of pregnancy. Key hormonal factors implicated in the regulation of insulin levels during pregnancy encompass placental prolactin, progesterone, prolactin, and estrogen. As gestation advances, the secretion of these hormones escalates progressively, resulting in diminished insulin sensitivity in peripheral tissues and a gradual rise in insulin resistance. Dysregulation of hormones in GDM patients results in prolonged elevation of blood glucose levels, leading to fetal hyperglycemia, hypertonic diuresis, and excessive amniotic fluid accumulation.^12^ These complications may predispose to preterm labor, premature rupture of membranes, and adverse fetal and perinatal outcomes.^13^ These studies indicate that increased insulin resistance in late pregnancy is associated with adverse pregnancy outcomes.

PROM in patients with GDM presents a significant threat to the well-being and viability of both mothers and infants. This study undertook a retrospective analysis of serum progesterone levels, HbA1c, insulin levels, and insulin resistance in GDM patients with and without PROM to assess the likelihood of PROM development in this population. The aim of this investigation is to ascertain the efficacy of these biomarkers in forecasting the risk of PROM in patients with GDM.

## Materials and methods

### Study subjects

**Fig. 1:**
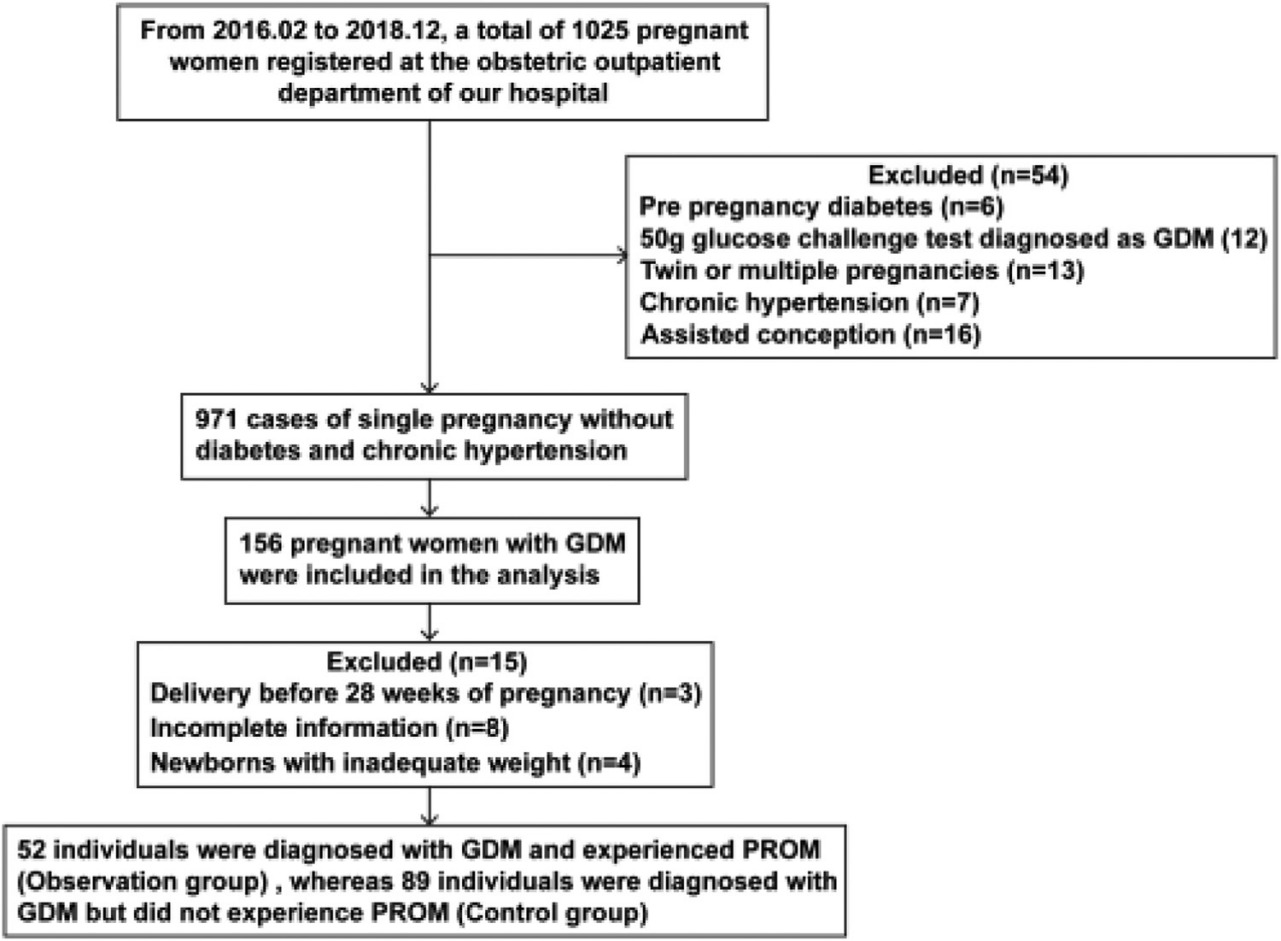
Chart for the selection of participants.

This study follows the STROBE reporting case-control guidelines. The present study was a retrospective study conducted in accordance with the Declaration of Helsinki. All subjects involved in the study gave informed consent. For this case-control study, 141 women with GDM were diagnosed and treated in the obstetrics outpatient clinic of Sinopharm Dongfeng General Hospital Affiliated with Hubei University of Medicine from February 2016 to December 2018, comprising 52 women with a diagnosis of GDM and PROM (defined as the observation group in this study) and 89 women with a diagnosis of GDM but without PROM (defined as the control group) (Fig. 1). All women were not diagnosed with diabetes mellitus before 24 weeks of gestation and underwent a 75g Oral Glucose Tolerance Test (OGTT) between 24 and 28 weeks of gestation. The diagnostic criteria for GDM were performed according to the criteria proposed by the International Association of Diabetes and Pregnancy Groups (20587717). PROM diagnosis was made according to previously published criteria,^14^^,^^15^ where gestational age < 37 weeks was defined as preterm-PROM (pPROM), and gestational age ≥ 37 weeks was defined as term-PROM (tPROM). Inclusion criteria were as follows: (1) GDM [Fasting Plasma Glucose (FPG) ≥ 5.1 mmoL/L (92 mg/dL) or 1h plasma glucose ≥ 10.0 (180 mg/dL) or 2 h plasma glucose ≥ 8.5 (153 mg/dL)] diagnosed at 24‒28 weeks of gestation; (2) Age 18‒38 years; (3) Consciousness and normal thinking; (4) Natural conception.

Exclusion criteria: (1) Twin or multiple pregnancies; (2) Chronic hypertension; (3) Women who delivered before 28 weeks; (4) Diagnosis of pre-GDM, i.e., glycemic profile at the time of the first pregnancy test, with an FPG ≥ 7.0 mmoL/L, an HbA1c ≥ 6.5 %, and/or a randomized glucose ≥ 11.1 mmoL/L; (5) Diagnosis of GDM only via a 50g Glucose Screening Test (GCT), FPG ≥ 5.3 mmoL/L (95 mg/dL), or 1 h plasma glucose ≥ 10.0 mmoL/L (180 mg/dL) or 2 h plasma glucose ≥ 8.6 mmoL/L (155 mg/dL) or 3 h plasma glucose ≥ 7.8 mmoL/L (140 mg/dL).

### General patient information

Obstetricians collected basic information about patients, including age, pre-pregnancy weight, height, number of deliveries, glycemic control, weight at the time of diagnosis of GDM, weight at delivery, mode of delivery, gestational weeks, adverse perinatal outcomes including puerperal infections and postpartum hemorrhage, as well as neonatal weight and Clinical Risk Index for Baby (CRIB) score. Pre-pregnancy weight and height were collected by pre-pregnancy examination or at the first pregnancy examination. The weight at delivery was collected at the time of admission to delivery. Postpartum hemorrhage refers to the total amount of bleeding more than 500 mL in vaginal delivery and more than 1000 mL in cesarean section within 24h after delivery. The basic information about the newborn was recorded immediately. Pregnancy outcome data and information were obtained from electronic health records.

### GDM prenatal care

All GDM women were referred to the health management center for GDM prenatal care, including health education, psychological care, diet, and exercise guidance. Following a 15-day nursing education program, venous blood samples were collected to assess Fasting Blood Glucose levels (FBG), prompting the nurses to make necessary adjustments to their diet and activity regimen. GDM women self-monitored blood glucose before and 2h after meals through a home blood glucose meter. The target range of blood glucose control is: FBG is less than 5.3 mmoL/L, 2 h postprandial blood glucose is less than 6.7 mmoL/L. According to the patient's review results and self-reported blood glucose values, when the blood glucose exceeds the target range, blood glucose control is defined as poor, and insulin treatment is required. Monitoring blood glucose control should continue until the pre-production phase.

### Laboratory measurements

All subjects were fasted for 12h, and peripheral venous blood was taken in the early morning of the next day in the first trimester (6‒12 weeks), the second trimester (24‒28 weeks), and the third trimester (32 weeks). The blood samples were placed at 4°C for 2h, and then centrifuged at 3000g for 15 minutes using a centrifuge. The supernatant was collected as serum. In early pregnancy, Total Cholesterol (TC) and Triglyceride (TG) were measured in all samples. Fasting glucose, fasting insulin, glycosylated Hemoglobin (HbA1c), and progesterone levels were measured in the second and third trimesters of pregnancy. Blood glucose levels were measured at 1h and 2h after taking sugar; insulin levels were measured at 1h, 2h and 3h after taking sugar. An automatic biochemical analyzer (Roche Cobas 6000) was used to measure blood glucose, HbA1c, TC and TG. Roche Modular Analytics E170 (Roche) was used for the electrochemical immunoluminescence assay of insulin. Homeostasis model assessment (HOMA) was used to assess insulin resistance (HOMA-IR) and islet β-function index (HOMA-β) in GDM. HOMA−IR=fastingplasmainsulinconcentration(μU/mL)×FBG(mmoL/L)/22.5.HOMA−β=20×fastingplasmainsulinconcentration(μU/mL)/(FBG[mmoL/L]−3.5).

### Sample size calculation

The sample size of the study was estimated using G*Power software version 3.1.9.2, with a significance level of α = 0.05, power to 1- β = 0.8, effect size *d* = 0.7, and bilateral testing. The results indicated that each group should have a minimum of 40 to 48 samples, based on the binary logic analysis with 5 to 6 independent variables requiring a sample size 8 to 12 times that of the independent variables. In the study, there were 52 cases in the observation group and 89 cases in the control group.

### Statistical analysis

SPSS 20.0 software was used for statistical analysis. The Shapiro-Wilk test was used to determine the normality of the data. The measurement data were expressed as mean ± standard deviation. For continuous variable data in a normal distribution, a *t*-test was used for inter-group comparison, and the Mann-Whitney *U* test was used for continuous variable data in a skewed distribution. Count data were expressed as frequencies and ratios and were tested by Chi-Square or Fisher's exact test. Two independent samples were used for the non-parametric test of between-group analysis. Comparative analysis was performed by the Chi-Square test. The difference was statistically significant (p < 0.05). The ROC curve and AUC were utilized to assess the predictive accuracy of the indicators for PROM in patients with GDM. In addition, logistic regression analysis was conducted to identify potential risk factors for predicting the risk of PROM and adverse perinatal outcomes in GDM by examining statistically significant parameters between groups.

## Results

### General clinical information and risk factors of PROM in GDM pregnant women

As shown in Table 1, a total of 141 patients with GDM were included in the study. There was no significant difference in age, pre-pregnancy BMI (too light or overweight), number of deliveries, and blood lipid level between the control group and the observation group (p > 0.05). However, the authors found that patients in the observation group had higher FPG (p < 0.001) and lower FINS (p = 0.016). In addition, the blood glucose control in the observation group was poor and the GWG was significantly higher than that in the control group (p < 0.05). By stepwise binary Logistic analysis (Table 2), poor blood glucose control and high GWG were potential risk factors for PROM in GDM patients.

**Table 1:** General clinical data of participants.

**Characteristic**	**Observation group** **(n** = **52)**	**Control group** **(n** = **89)**	**p-value**
Age (years)	32.51 ± 5.37	31.92 ± 4.34	0.470
Pre-pregnant BMI (kg/m^2^)			0.077
< 18.5	9	20	
> 24.0	7	24	
FINS (mU/L)	11.06 ± 1.34	11.62 ± 1.32	0.016
FPG (mmoL/L)	6.07 ± 0.58	5.33 ± 0.68	< 0.001
Parity			0.934
Primiparity	40	69	
Multiparity	12	20	
Glycemic control			0.005
Good	38	81	
Poor	14	8	
Serum Lipid			
TC (mmoL/L)	4.23 ± 0.59	4.36 ± 0.73	0.362
TG (mmoL/L)	1.08 ± 0.73	1.12 ± 0.62	0.485
GWG (kg)	12.46 ± 3.27	10.98 ± 3.56	0.005

GWG, Calculate the total weight gain during pregnancy by subtracting the weight before pregnancy from the weight at delivery. 28‒36+6: gestational age 28‒36 weeks+6 days.

**Table 2:** Multiple stepwise regression analysis between PROM and various indicators.

**Variable**	**OR (95** % **CI)**	**p-value**
Glycemic control	1.59 (1.257‒1.934)	< 0.001
GWG (kg)	1.12 (1.097‒1.529)	0.016

Note: OR, Odds Ratio; CI, Confidence Interval.

### Perinatal and neonatal outcomes

**Fig. 2:**
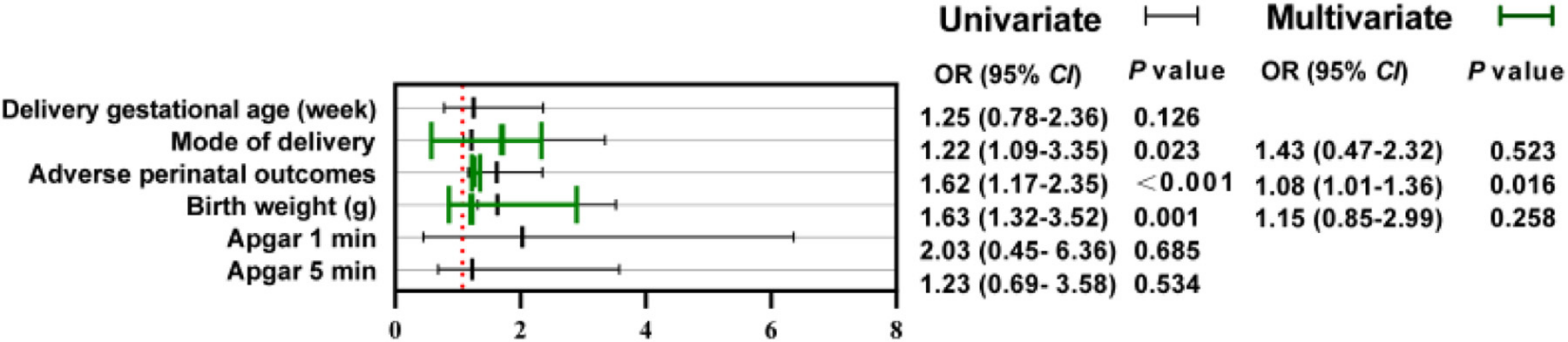
The impact of PROM on pregnancy outcomes and newborns in GDM pregnant women.

Next, the authors analyzed the perinatal outcomes and neonatal outcomes of the two groups, as shown in Table 3. The authors found that compared with the control group, the observation group had a higher proportion of cesarean section (p < 0.001), younger delivery gestational age (p = 0.013), and a higher incidence of postpartum puerperal infection and postpartum hemorrhage (p = 0.019). Subsequently, the present study examined the weight of two cohorts of newborns, stratifying them into subgroups according to the median weight gain during pregnancy (GWG = 11.69 kg). Analysis revealed that newborns in the observation group with GWG below 11.69 kg exhibited significantly lower weights compared to those in the control group (p < 0.001). Conversely, newborns with GWG exceeding 11.69 kg displayed slightly higher weights than the control group, although this disparity was not statistically significant (p > 0.05). In addition, the authors found no significant difference in neonatal PGAR scores at 1 min or at 5 min between the two groups (p > 0.05). Univariate and multivariate logistic analysis showed that PROM significantly increased the adverse pregnancy outcomes of GDM pregnant women, including puerperal and postpartum hemorrhage (OR = 1.08, 95 % CI 1.01‒1.36, p = 0.016), as shown in Figure 2.

**Table 3:** Perinatal and neonatal outcomes of pregnant women.

**Characteristic**	**Observation group** **(n** = **52)**	**Control group** **(n** = **89)**	**p-value**
Delivery gestational age (week)	36.14 ± 4.45	38.12 ± 3.77	0.013
Mode of delivery			
Categorized	22 (42.31)	12 (13.48)	< 0.001
Vaginal	30 (57.69)	77 (86.52)	
Adverse perinatal outcomes			0.019
Puerperal infection	7 (13.46)	3 (3.37)	
Postpartum haemorrhage	5 (9.62)	2 (2.24)	
Birth weight (g)			
GWG < 11.69 kg	2559.03 ± 322.73	3250.47±359.39	< 0.001
GWG ≥ 11.69 kg	3324.18 ± 455.25	3209.7±413.27	0.238
PGAR scores			
Apgar 1 min	8.69 ± 1.05	8.86 ± 0.73	0.451
Apgar 5 min	8.9 ± 0.91	9.19 ± 0.89	0.069

Apgar 1/5 min: PGAR scores at 1 min or at 5 min.

### The predictive efficacy of HbA1 c and HOMA-IR for PROM in GDM patients

**Fig. 3:**
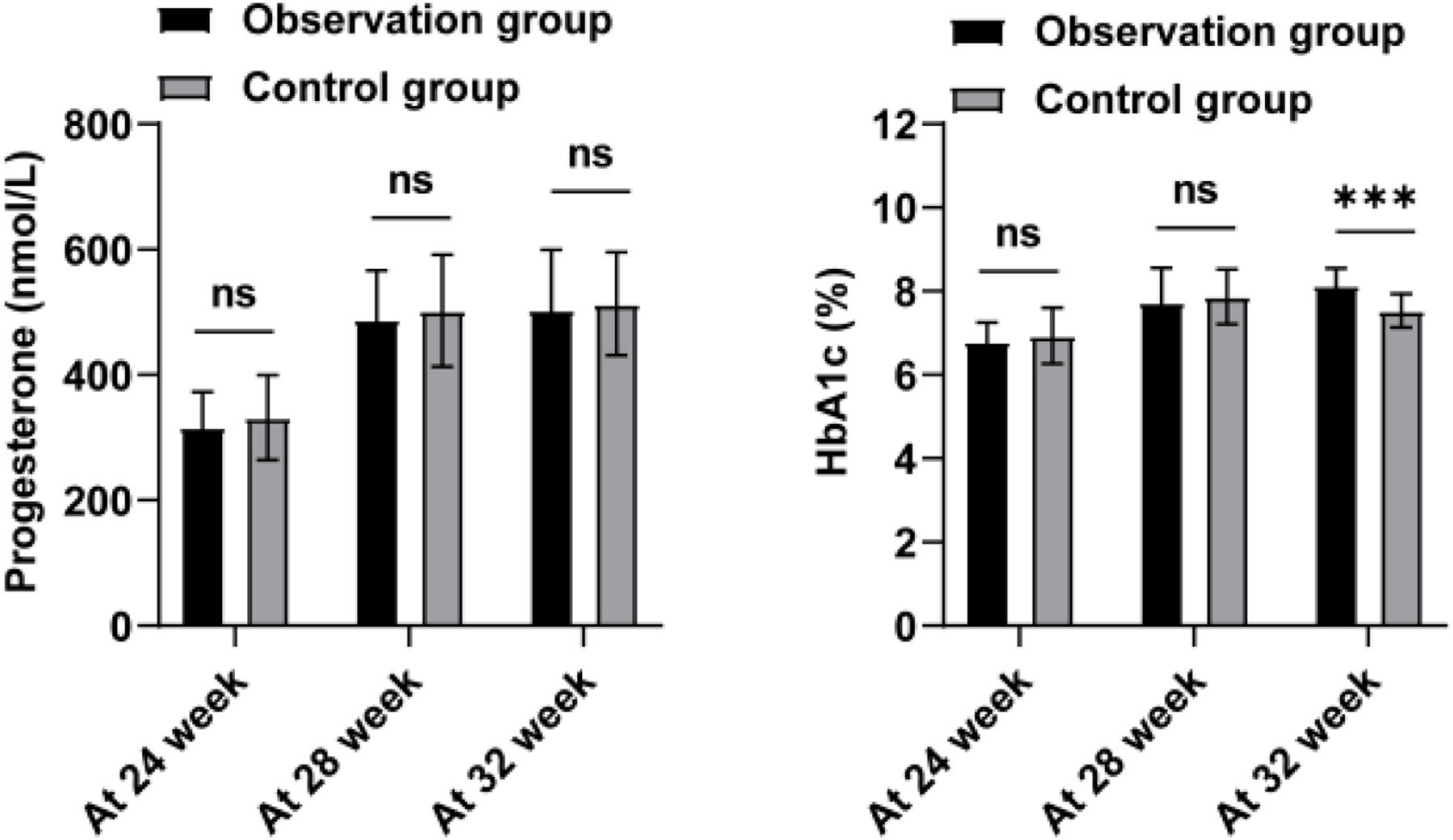
Changes progesterone and HbA1c levels in serum of patients in the two groups.

**Fig. 4:**
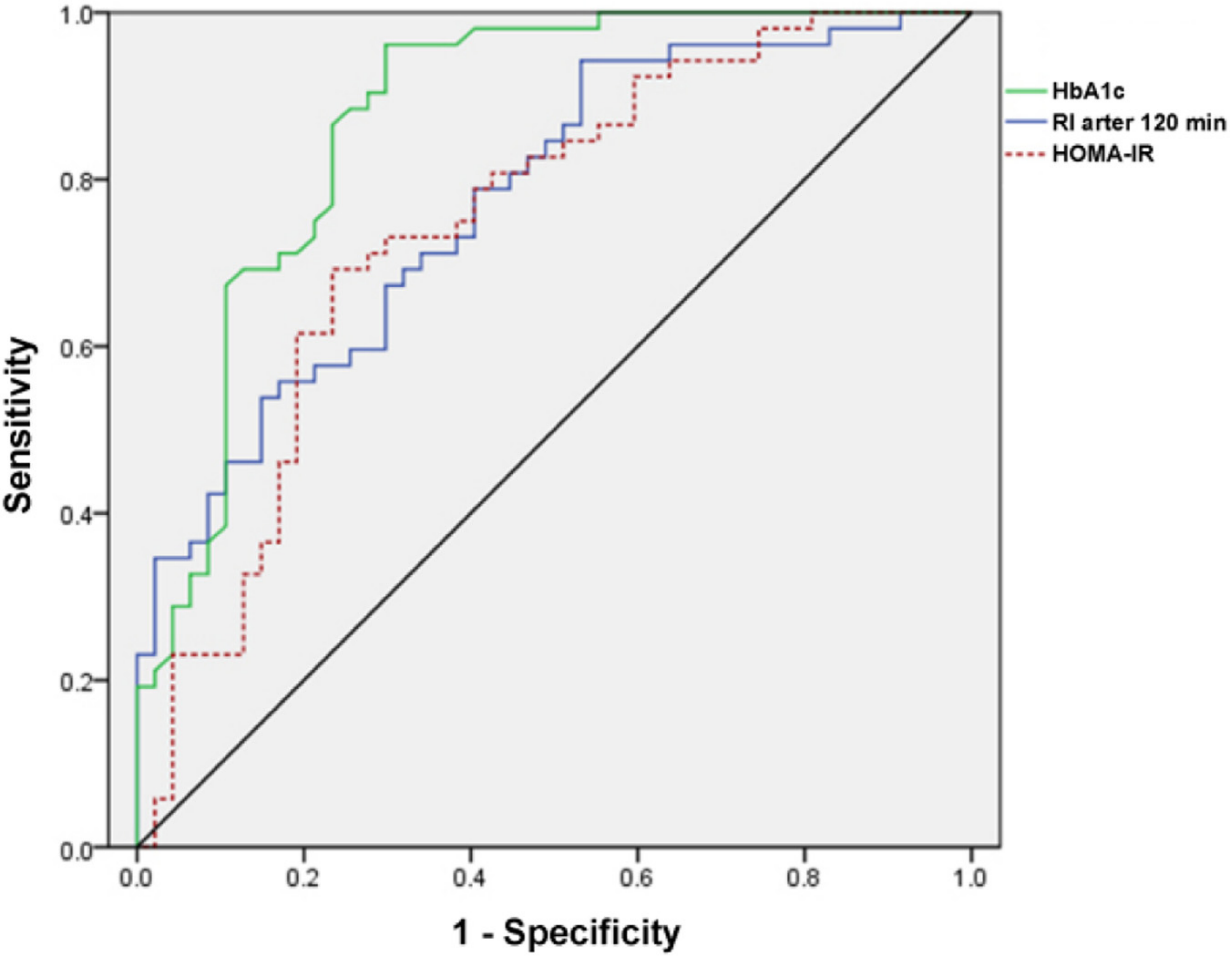
ROC curve of predictive efficacy of HbA1c, RI after 120 min, and HOMA-IR for PROM.

Next, the authors analyzed the progesterone and HbA1c levels of the two groups of patients at 24, 28, and 32 weeks, as shown in Figure 3 and Table 4. Progesterone levels did not show a notable discrepancy between the two groups during each time interval (p > 0.05). Only at 32 weeks of gestation, the HbA1c level in the observation group was significantly higher than that in the control group (p < 0.001). Further, the authors analyzed the insulin levels and insulin resistance in the two groups (Table 4). Normally, insulin secretion peaks 30‒60 min after a glucose load and then begins to decrease. The authors observed that the peak period of insulin was 120 min after glucose load, and the degree of insulin reduction in the observation group was lower than that in the control group (p = 0.022). HOMA-IR of the observation group was higher (p = 0.008). This indicates that the patients in the observation group have higher insulin resistance. To further explore the valuable application of HbA1c, RI after 120 min, and HOMA-IR in predicting the occurrence of PROM in GDM patients, the authors established ROC curves (Table 5 and Fig. 4). According to the ROC curve analysis, the AUC of HbA1c for PROM in GDM patients was 0.873 ± 0.037, with a critical value of 7.695; the AUC of glucose loading after 120 min was 0.774 ± 0.046, with a critical value of 157.59 (mU/L); the AUC of HOMA-IR was 0.747 ± 0.050, with a critical value of 2.802 (nmoL/L). These results suggest that HbA1c, RI after 120 min, and HOMA-IR are better able to separate patients with and without PROM in GDM patients.

**Table 4:** Progesterone, HbA1c, and insulin resistance levels in the groups.

**Index**	**Observation group** **(n** = **52)**	**Control group** **(n** = **89)**	**p-value**
HbA1c (%) at 32 week	8.09 ± 0.41	7.53 ± 0.52	< 0.001
RI after 60 min (mU/L)	95.86 ± 13.14	96.89 ± 11.41	0.435
RI after 120 min (mU/L)	169.22 ± 9.77	155.99 ± 7.18	0.022
RI after 180 min (mU/L)	50.97 ± 1.33	47.55 ± 1.78	0.190
HOMA-IR	2.97 ± 0.41	2.77 ± 0.43	0.008
HOMA-β	155.35 ± 88.34	150.93 ± 104.04	0.80

RI: Insulin, RI after 60/120/180 min means insulin levels after taking 75g of glucose for 60, 120, and 180 minutes.

**Table 5:** Predictive efficacy of HbA1c, RI after 120 min, and HOMA-IR for premature rupture of membranes.

**Parameter**	**AUC**	**Critical value**	**Sensitivity**	**Specificity**
HbA1c (%)	0.873 ± 0.037	7.695	0.962	0.702
RI after 120 min	0.774 ± 0.046	157.59	0.892	0.468
HOMA-IR	0.747 ± 0.050	2.802	0.692	0.766

## Discussion

There is a deficiency in reliable predictors and preventive strategies for preterm PROM in patients with GDM. The current investigation explores the relationship between GDM and PROM, offering insights into the prediction and prevention of PROM in the clinical management of GDM. While prior research has primarily concentrated on individual risk factors for either GDM or PROM, limited attention has been given to the combined risk factors for GDM and PROM. This retrospective study demonstrates the predictive value of HbA1c levels at 32 weeks, insulin levels after 120 minutes of glucose loading, and HOMA-IR levels in identifying the onset of PROM in GDM patients.

Currently, there are fewer studies related to the theory of sex hormones in GDM in China, and the specific mechanism of progesterone's role in GDM needs to be further studied in depth.^16^ In this study, the authors did not find any difference in progesterone in GDM women with and without combined PROM. HbA1c is a commonly used clinical indicator for blood glucose detection, especially playing an important role in the screening and diagnosis of GDM in early pregnancy.^17^ Several studies have shown that patients with GDM have significantly elevated blood glucose and HbA1c compared with healthy pregnant women in pregnancy, and the incidence of PROM in patients with GDM is higher than that in healthy women.^18^ In a prior investigation, Guo et al. examined 22 pregnant women with GDM in conjunction with preterm PROM during mid-pregnancy (24 to 28 weeks) and found no significant elevation in FINS and FPG levels compared to those with GDM who did not present PROM.^19^ Contrary to these findings, this study revealed elevated levels of FINS and FPG in GDM who presented PROM. The discrepancy in findings between the two studies may be attributed to the limited sample size of the prior investigation. In a survey of a pregnant population in Shenzhen, increased FBG in early pregnancy is associated with increased risk of cesarean section, macrosomia, and PROM.^20^ In this study, the authors observed that at 32 weeks of gestation, HbA1c levels in GDM patients with PROM were significantly higher than those in patients without PROM. HbA1c can reflect the average blood glucose level within 8‒12 weeks, so it is also commonly used in the evaluation of blood glucose control in diabetic patients.^21^ It is worth noting that the authors observed that patients with GDM combined with PROM had poor blood glucose control after diagnosis of GDM, which may explain the increase in HbA1c levels in this group of patients. Inadequate management of blood glucose levels during pregnancy is linked to negative outcomes for both the mother and the newborn.^22^^,^^23^ Studies have shown that blood glucose control at different time points in patients with GDM is related to pregnancy outcomes. Gestational age has a greater risk of abnormal blood glucose control in GDM patients, and good blood glucose control plays an important role in improving pregnancy complications and perinatal conditions.^24^ In another prospective study of blood glucose in pregnant women with 14-day continuous glucose monitoring GDM, poor blood glucose control or high continuous blood glucose levels are associated with adverse pregnancy outcomes, such as PROM.^25^ The present study also confirmed that poor glycemic control was a potential risk factor for PROM in GDM patients. ROC analysis also showed that HbA1c values of more than 7.695 % can better predict the risk of PROM in GDM patients. However, in this analysis, no difference in PGAR scores was observed between GDM with PROM and without PROM.

The insulin secretion during pregnancy is often severely limited, and impaired glucose tolerance is associated with β-cell dysfunction and insulin resistance.^26^ HOMA-IR and HOMA-β are steady-state models for evaluating insulin resistance and β-cell function.^27^ Increased HOMA-IR during the first trimester can increase the likelihood of developing GDM, and body weight has an impact on HOMA-IR levels.^28^ The degree of insulin resistance is associated with weight gain in GDM patients.^29^ Similarly, the present analysis showed that HOMA-IR and total pregnancy weight gain were higher in GDM patients with PROM, and ROC analysis showed that HOMA-IR exceeding the critical value of 2.802 could better distinguish GDM patients with PROM from GDM patients without PROM. In addition, the results of the logical analysis showed that GWG was confirmed to be associated with the risk of PORM in GDM patients. Although the increase in HbA1c levels in early pregnancy reflects β-cell dysfunction in GDM pregnant women,^30^ no difference in HOMA-β between the two groups was found in this analysis. The difference in results may be due to the detection of HOMA-β in the second trimester, while the results of the previous study were detected in early pregnancy. Delayed peak insulin is a risk factor for preeclampsia and neonatal hypoglycemia during pregnancy.^31^ In particular, the present analysis found that the insulin levels of the two groups peaked after 120 min of glucose loading, showing delayed insulin secretion, and did not decrease to fasting insulin levels after 180 min. It is worth noting that the insulin level after 120 min of glucose loading was higher in GDM patients with PROM, and 157.59 mU/L can better predict the risk of PROM in GDM.

However, this study was a single-center retrospective analysis, and the selection of research objects inevitably introduced bias. For instance, the limited sample size necessitates further examination of the presence of these characteristics in individuals with pre-pregnancy diabetes and early pregnancy hyperglycemia. The present study specifically targets women with GDM; thus, the findings may not be generalizable to non-GDM women. Due to the constraints of the sample size, this analysis did not account for potential confounding variables such as dietary habits, physical activity, smoking, and alcohol intake. In addition, the authors did not distinguish between pPROM and tPROM. Finally, it is necessary to design a prospective study to observe the changes in HbA1c, insulin, and HOMA-IR in patients before, during, and after pregnancy, and to clarify the relationship between these changes and PROM and pregnancy outcomes in GDM patients.

## Conclusion

In conclusion, the present study showed that HbA1c (at 32 weeks), insulin at 120 min after glucose loading, and HOMA-IR levels can effectively predict PROM in GDM patients and are associated with postpartum adverse pregnancy outcomes, such as puerperal infection and postpartum hemorrhage. In addition, poor blood glucose control and weight gain during pregnancy are independent risk factors for PROM in GDM patients.

## Availability of data and materials

The datasets used and/or analyzed during the present study are available from the corresponding author upon reasonable request.

## Ethics statement

All procedures performed in this study involving human participants were in accordance with the ethical standards of the institutional and/or national research committee and with the 1964 Helsinki Declaration and its later amendments or comparable ethical standards. All subjects were approved by the Sinopharm Dongfeng General Hospital Affiliated to Hubei University of Medicine (nº SDFGH201506).

## Author's contributions

LiRong Zhou and XueSong Xiong designed the research study. LiRong Zhou and LianHua Chen performed the research. XueSong Xiong and LianHua Chen provided help and advice. LiRong Zhou, XueSong Xiong and LianHua Chen analyzed the data. LiRong Zhou and XueSong Xiong wrote the manuscript. LianHua Chen reviewed and edited the manuscript. All authors contributed to editorial changes in the manuscript. All authors read and approved the final manuscript.

## Funding

Not applicable.

## Declaration of competing interest

The authors declare no conflicts of interest.
